# A novel non-segmented inverted water outline rendering method can improve the tracking of responsible blood vessels for hemifacial spasm

**DOI:** 10.3389/fnins.2024.1296019

**Published:** 2024-01-30

**Authors:** Li Zhenzhu, Zhang Jingfeng, Zhou Wei, Zheng Jianjun, Xia Yinshui

**Affiliations:** ^1^Radiology Department, Ningbo NO.2 Hospital, Ningbo, China; ^2^Department of Neurosurgery, Ningbo NO.2 Hospital, Ningbo, China; ^3^Faculty of Electrical Engineering and Computer Science, Ningbo University, Ningbo, China

**Keywords:** hemifacial spasm spasm, REZ, image, 3D Slicer, MVD

## Abstract

This study aimed to explore a novel, non-segmented based on inverted water outline, and rapid 3D rendering method for identifying the responsible blood vessels for hemifacial spasm. First, the software was developed using the free and open-source 3D Slicer to process magnetic resonance images. Outlines of the water region were extracted and rendered in a three-dimensional space. The traditional image re-slicing technique (IMRT) was used for the control group, while non-segmented inverted water outline rendering (NSIWR) was used to observe the relevant blood vessels in the root entry/exit zone (REZ) of patients with hemifacial spasm. The intraoperative exploration results were considered the gold standard for comparing the differences in identifying relevant blood vessels between the two methods. Twenty-five patients were included, and the reconstruction effect evaluation suggested that NSIWR could effectively reconstruct the responsible blood vessels of the cochlea, facial nerve, and REZ. Compared with IMRT, NSIWR effectively improved the diagnosis of the responsible blood vessels in the REZ, clarified their sources and directions, and was consistent with intraoperative results. This study introduced a novel rapid rendering method based on NSIWR, which was successfully applied for hemifacial spasm. The method enhances accuracy in identifying responsible blood vessels in the REZ without needing multi-modal techniques. It has the potential to improve surgical effectiveness and reduce exploration time in treating hemifacial spasm.

## 1 Introduction

Hemifacial spasm (HFS) are characterized by involuntary intermittent facial twitching that typically originate from the eyelid on the affected side (Lee et al., 2021). Over time, the symptoms can worsen and involve all muscles on the same side of the face, leading to difficulties in communication and mobility, such as limited driving vision (Cheng et al., 2015; Sindou and Mercier, 2018). The underlying cause of HFS is believed to be the compression of the facial nerve roots at the root entry/exit zone (REZ) by blood vessels (Cheng et al., 2015; Sindou and Mercier, 2018). Microvascular decompression (MVD) is considered the most effective treatment for HFSs and involves the isolation of blood vessels and nerves in the REZ. Compared with alternative treatment options, such as medications or botulinum toxin, MVD can offer complete relief from HFS symptoms (Sindou and Mercier, 2018). However, approximately 10% of patients do not respond well to the surgery, which may be attributed to the misidentification of the responsible blood vessels during the procedure (Dumot and Sindou, 2018; Sindou and Mercier, 2018).

Magnetic resonance imaging (MRI) is currently the preferred pre-operative imaging technique for HFSs because it can identify the offending vessels and nerves (Docampo et al., 2015; Han et al., 2021). The accurate identification of the responsible blood vessels in a two-dimensional plane view requires tracing multiple layers of images, which is time-consuming and labor-intensive (Hughes et al., 2021). However, some researchers have employed a manual segmentation approach to reconstruct three-dimensional images after segmenting blood vessels and nerves (Zhou et al., 2012; Dolati et al., 2015). Although this segmentation method exhibits high sensitivity and specificity for detecting responsible blood vessels, it is time-consuming (Guo et al., 2012; Haller et al., 2016).

Our research team discovered that the MRI water signal revealed both the facial nerves and corresponding blood vessels. Consequently, we hypothesized that contouring the water signal could enable the observation of the relationship between blood vessels and nerves. Building on this concept, we employed grayscale reversal to enhance the low-intensity water medium in MRI. Subsequently, we used contouring and fast 3D rendering technology to visualize the blood vessels responsible in the REZ. This novel imaging technique not only facilitates the rapid elimination of non-water soft tissue interference but also enhances the clinical diagnosis of HFSs.

Therefore, in this study, we developed a plugin to achieve this functionality using the free and open-source software, 3D Slicer. We aimed to automate and streamline the process of three-dimensional rendering of nerves and blood vessels in the REZ. We used the traditional image re-slicing technique as a control group and intraoperative exploration as the gold standard.

## 2 Materials and methods

### 2.1 Study design and participants

**Figure 1:**
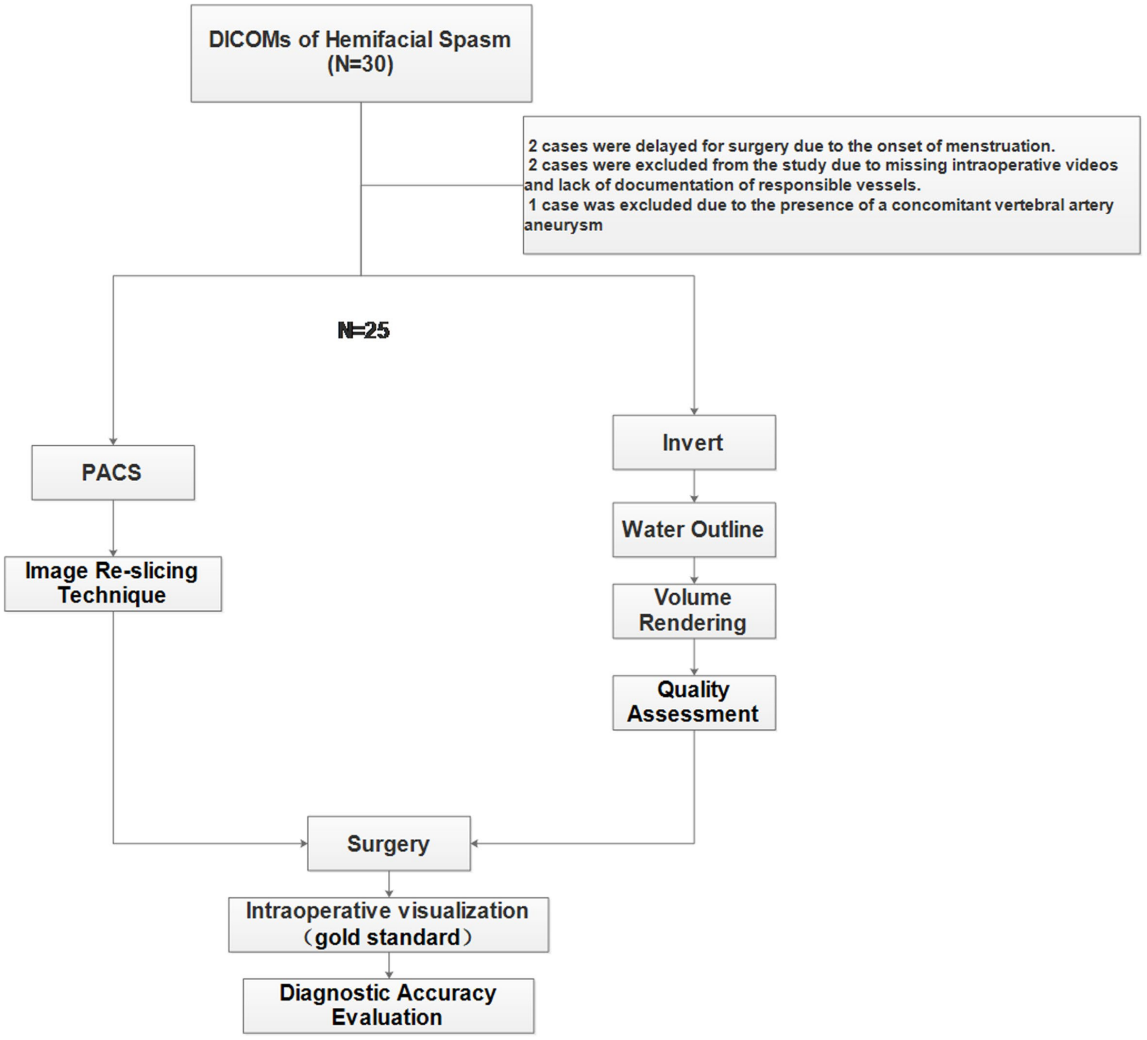
The flowchart of the study.

This study was approved by the Ethics Committee of Ningbo NO.2 Hospital in accordance with the revised World Medical Association Declaration of Helsinki and followed the Strengthening the Reporting of Observational Studies in Epidemiology (STROBE) statement guidelines. All patients were strictly screened according to the inclusion and exclusion criteria (Figure 1). The inclusion criteria were a diagnosis of facial spasm, an MR facial nerve scan, and surgical treatment at our hospital. The exclusion criteria were the presence of concomitant diseases, such as facial herpes zoster or facial paralysis, and treatment with botulinum toxin injection.

From April 2021 to November 2022, we prospectively enrolled patients (*N* = 30) with hemifacial spasm. All patients underwent advanced MRI examinations, including 3D fast spin echo T2-weighted imaging (T2WI). The diagnoses of all patients were made by the same physician (Li Zhenzhu, Clinical work experience of more than 10 years). All patients underwent both a traditional diagnosis (IMRT) based on the PACS and a diagnosis based on the non-segmented inverted water outline rendering technique (NSIWR) using the 3D Slicer platform. The final vascular and neural relationships were determined through intraoperative observations. Experienced neurosurgeons (Zhou Wei, Clinical work experience of more than 25 years) performed MVD.

### 2.2 MRI scanning and image data acquisition

The MR examination included a routine protocol for brain examination (Supplementary File 1). Philips has a sequence called driven-equilibrium spin-echo (DRIVE), which is a 3D TSE technique that produces high-resolution T2-weighted images. The DRIVE pulse enables shorter TRs, making the 3D TSE method faster. Owing to its lower sensitivity for flow voids compared to multi-slice sequences, 3D DRIVE is particularly useful for improving fluid visualization in imaging the internal auditory canal.

### 2.3 Image re-slicing technique

After completing the MRI scan, the data were automatically uploaded to the PACS. The doctors used the software provided by the system to re-slice along the facial nerve, including reconstruction of the sagittal and coronal planes. They described the relationship between blood vessels and nerves, traced the source of blood vessels through slicing, and ultimately reported on nerves and blood vessels that may have caused compression. They determined the side of the convulsions using the medical record system.

### 2.4 Non-segmented inverted water outline rendering

**Figure 2:**
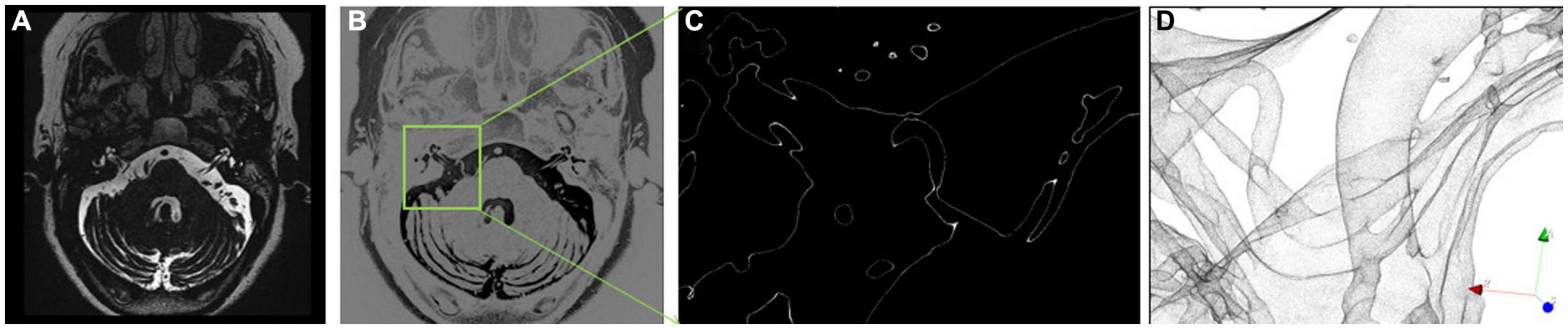
Non-segmented inverted water outline technique. **(A)** MR image, **(B)** inverted grayscale image; **(C)** extraction of water outline within ROI; **(D)** three-dimensional rendering based on water outline.

The method (Supplementary Video) was implemented using the free and open-source 3D Slicer software, and all steps were completed within 1–3 min. The specific approach initially involved performing a gray-scale inversion on the image data obtained from the drive sequence (Figure 2A). The inverted image was then truncated and rendered based on a water threshold (Figures 2B,C). Subsequently, a square region of interest (ROI) was selected to adjust the cropping of the affected side’s REZ in the orbit to the center. The 3D rendering image was observed in the 3D window with rotation and translation. The cropped 3D image included the ipsilateral cochlea, as it could help in quickly determining the position of the facial nerve. Observations were made in the direction of the ventral aspect of the brainstem to check for blood vessels passing through the REZ of the facial nerve root (Figure 2D).

### 2.5 Image of NSIWR quality assessment

The quality of the 3D reconstructed images was assessed to determine the optimal diagnostic effectiveness. The image quality was categorized as poor (score = 0), good (score = 1), or excellent (score = 2). The evaluation focused on essential anatomical structures, including the cochlea, facial nerve, REZ of the brainstem, vertebral artery, and posterior inferior cerebellar artery (PICA)/anterior inferior cerebellar artery (AICA) branches, encompassing five criteria. A combined assessment was performed by a neurosurgeon (Li Zhenzhu, Clinical work experience of 14 years) and a neuroradiologist (Zhang Jingfeng, Clinical work experience of more than 15 years). In case of any discrepancies in the scores, a thorough discussion was conducted between the two observers to reach a consensus score.

### 2.6 Surgery and intraoperative diagnosis

The surgery was performed by the same team with the aid of a microscope or an endoscope. All surgeries were performed using the retrosigmoid intradural approach. After successful tracheal intubation under general anesthesia, the patient was placed in a prone position on the right/left side, and the surgical field was routinely disinfected and covered with drapes. Continuous electromyography monitoring and triggered electromyography revealed delayed responses, indicating abnormal facial nerves.

After opening the skull through the left/right retrosigmoid approach, the skin-muscle flap was incised, and the bone flap was freed, exposing the sigmoid and transverse sinus corners with a bone window of approximately 3.5 cm × 3 cm. The dura mater was cut, the lower brainstem was opened to reduce the intracranial pressure, the left/right cerebellar hemisphere was dissected and detached from the arachnoid, and the facial nerve was exposed. The arterial segments and branches compressing the brainstem were marked and separated from the facial nerve with felt pads. After confirming that there was no compression, monitoring of the electromyography showed that the delayed waves disappeared. The bleeding was stopped, the dura mater was tightly sutured, and the cranial bone defect was repaired. The muscles and skin were sutured layer by layer. The surgical records documented the responsible blood vessels explored in the REZ and the source of the blood vessels during surgery.

### 2.7 Diagnostic accuracy evaluation for IMRT and NSIWR

Two experts (LZZ, ZJF) evaluated the anatomical relationship of the HFS side. The relationship between the vessels and nerves observed during surgery was used as the gold standard for diagnosing and evaluating the responsible vessels. Moreover, the origin of the responsible vessels was tracked as much as possible during surgery to identify the vessels. In all the groups, unidentified small responsible vessels or vessels with unknown sources were represented by anonymous arteries. If the visualization of the responsible vessels and affected nerves was consistent with the intraoperative view, the result was scored as 1; otherwise, it was scored as 0.

### 2.8 Statistical analysis

The measurement data for the normal distribution are presented as means ± standard deviations, and the comparison between groups was conducted using two independent sample t-tests. The measurement data for skewed distribution are presented as medians and quartile ranges. Categorical data are presented as the number of cases and their respective rates. Comparisons between groups were performed using the chi-squared or Fisher’s exact test. The level of statistical significance was set at *p* < 0.05. All statistical analyses were performed using the GraphPad software (version 7).

## 3 Results

### 3.1 Clinical characteristics of the participants

Thirty patients were enrolled in this study. However, five patients were excluded: two owing to delayed surgery caused by menstrual cycles, two others owing to unclear responsible vessels in the intraoperative video and postoperative medical records, and one because of the presence of a co-existing vertebral artery aneurysm. Ultimately, 25 patients were included in this study, and self-controlled comparisons were performed (Supplementary File 1). In the experimental group, the NSIWR technique was used to locate the responsible vessels in the REZ. In contrast, in the control group, the image re-slicing technique (IMRT) was used to identify the vessels (Table 1).

**Table 1:** Characteristics of the hemifacial spasm populations.

Clinical characteristics	
Age (years), median (IQR)	54 [38,74]
Male (male/female)	11/14
Site (L/R)	9/16
Spasm duration (years)	2.5 [0.4,10]

### 3.2 Evaluation of the quality of NSIWR

The quality was evaluated based on image diagnosis and important anatomical structures during surgery, including the cochlea, facial nerve, REZ of the brainstem, vertebral artery, and PICA/AICA branches, with a total of five items. The results showed that high scores were achieved for all the items evaluated (Supplementary File 1). Based on the reconstruction results, the cochlea was the most easily distinguishable (excellent: 92%), and the facial nerve (excellent:88%) could be traced along the cochlea.

### 3.3 Consistency with intraoperative images

**Figure 3:**
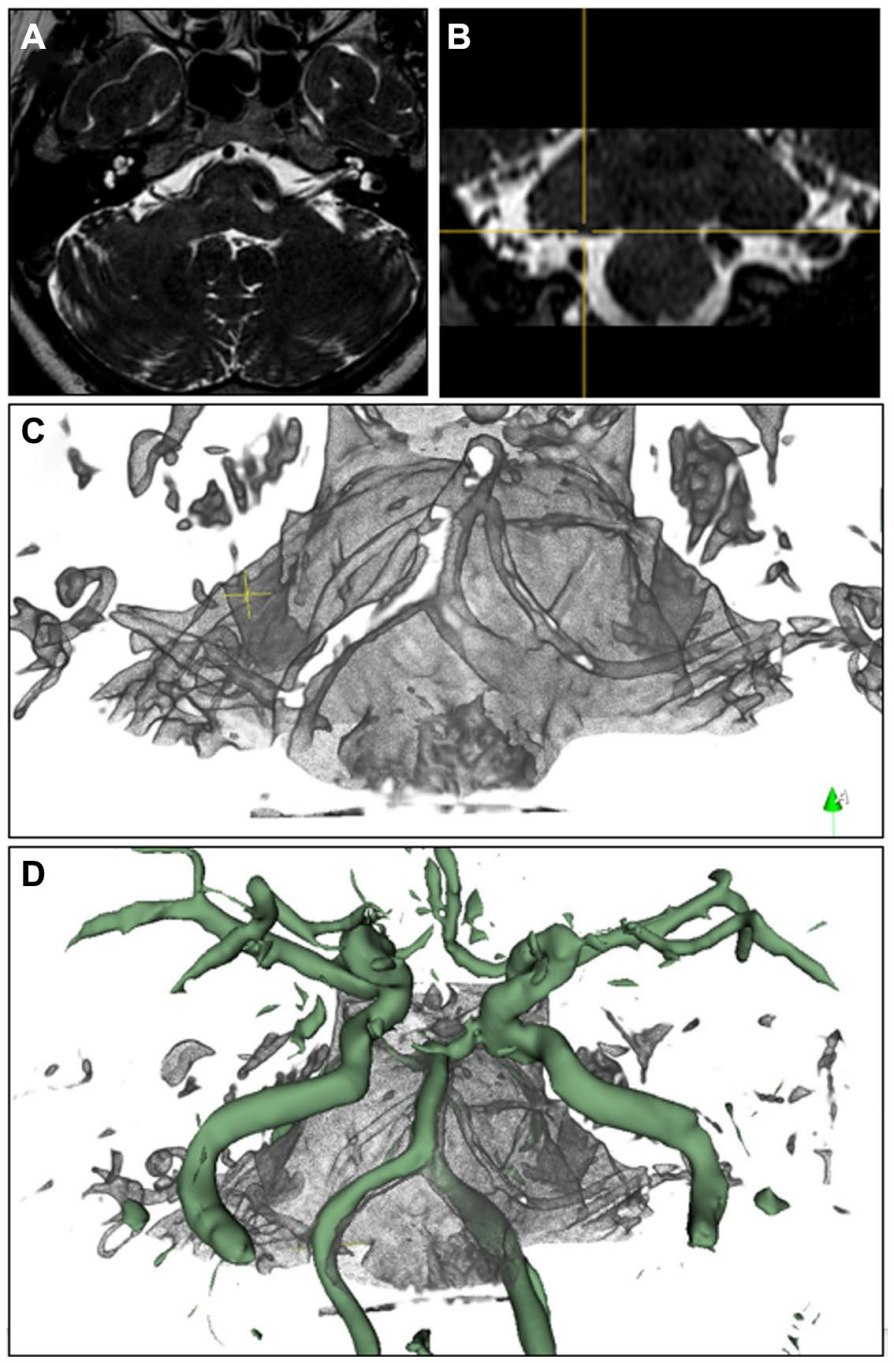
Rendering image of NSIWR. **(A,B)** MR images, **(C)** NSIWR in 3d space, **(D)** NSIWR and fusion with MRA reconstruction. The green vessels are the segmented reconstructions from MRA.

Among all patients with hemifacial spasm, NSIWR demonstrated significantly better results than the IMRT (*p* = 0.023). We performed subgroup analysis for cases involving single and multiple vessels, which revealed that the main advantage of NSIWR was its ability to effectively display the ROI, even when compressed by multiple vessels (88.9% vs. 11.1%, *p* = 0.015). The detailed scores obtained from the analysis are presented in Table 2. We also compared the reconstruction effects of NSIWR on small blood vessels with some MRA data (Supplementary Video), and found that NSIWR is significantly superior to MRA (Figure 3).

**Table 2:** Diagnostic accuracy of two methods.

	Intraoperative consistency	Intraoperative inconsistency	*p*-value
IMRT	17 (68.0%)	8 (32.0%)	
NSIWR	24 (96.0%)	1 (4.0%)	0.023
**Single culprit vessel (16)**			
IMRT	15 (93.7%)	1 (6.3%)	
NSIWR	16 (100.0%)	0 (0.0%)	>0.999
**Multiple culprit vessel (9)**			
IMRT	2 (22.2%)	7 (77.8%)	
NSIWR	8 (88.9%)	1 (11.1%)	0.015

### 3.4 Typical cases

#### 3.4.1 Case 1: Multiple vessels

**Figure 4:**
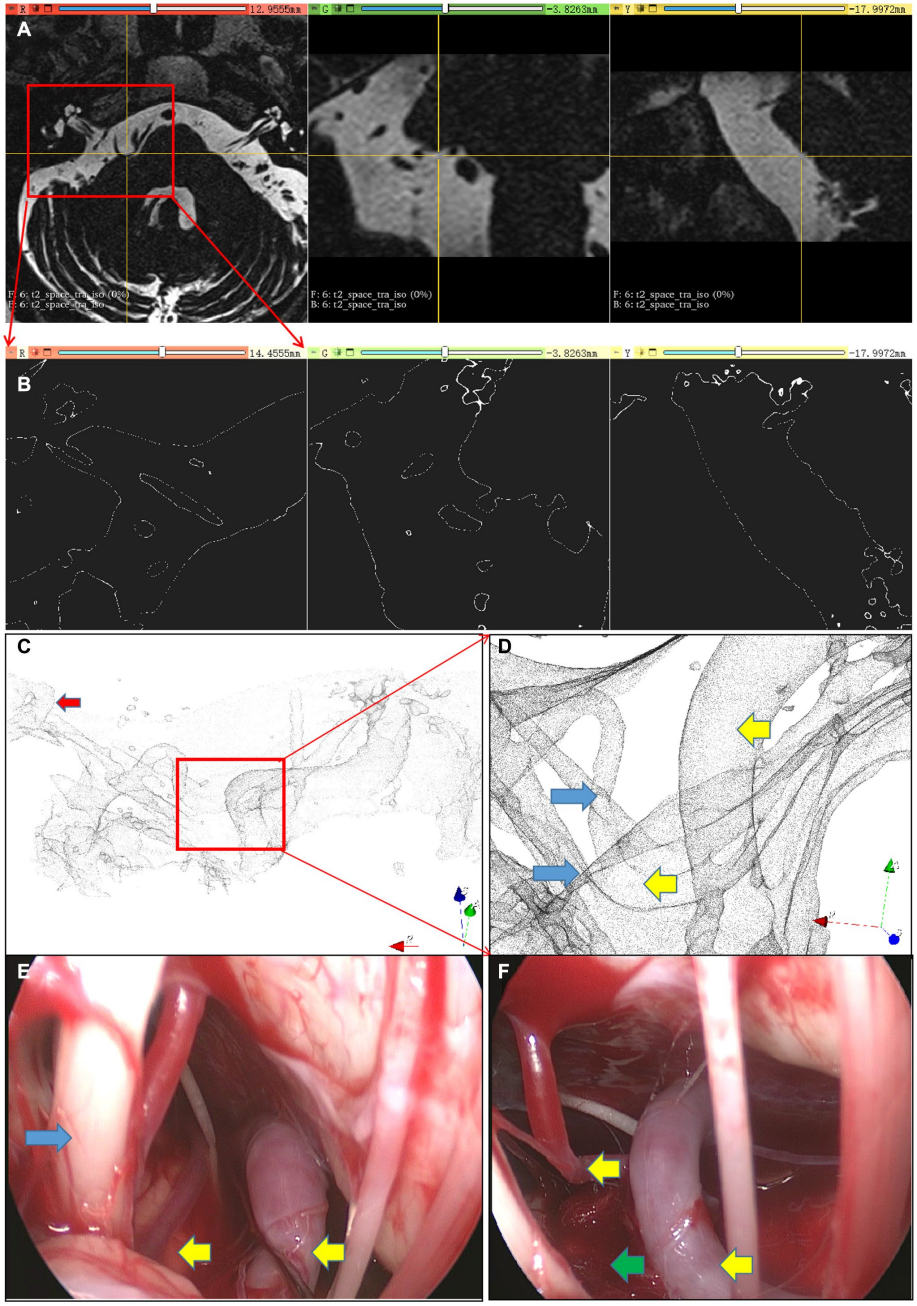
Multiple responsible vessel image. **(A)** Re-sliced image of the original image, **(B)** Extraction of inverted water outline, **(C)** three-dimensional rendering scene, **(D)** vessels and nerves within the REZ area; **(E,F)** microscopic images; Red arrow, Cochlea; Yellow arrow, Responsible vessel; Blue arrow, Facial nerve complex; Green arrow, Artificial material isolating vessels and nerves.

The patient experienced an unprovoked spasm on the right side of the face for 3 years despite previous treatment with intramuscular botulinum toxin. Pre-operative MRI (Figure 4A) revealed a close relationship between the right facial nerve and multiple blood vessels, including the vertebral artery and PICA. Using the inverted water outline technique (Figure 4B), important vascular and neural structures were observed across the three image sections. Subsequently, a three-dimensional rendering was produced to illustrate the cochlea and facial nerve, which could be traced toward the REZ, revealing the three-dimensional course of the vertebral artery and AICA within the REZ (Figures 4C,D). Under endoscopic guidance via the occipital sinus route, the surgery isolated all blood vessels within the REZ (Figures 4E,F), allowing for successful treatment after the removal of the compression from the vertebral artery and AICA. The patient did not experience any further hemifacial spasm postoperatively.

#### 3.4.2 Case 2: single vessel

**Figure 5:**
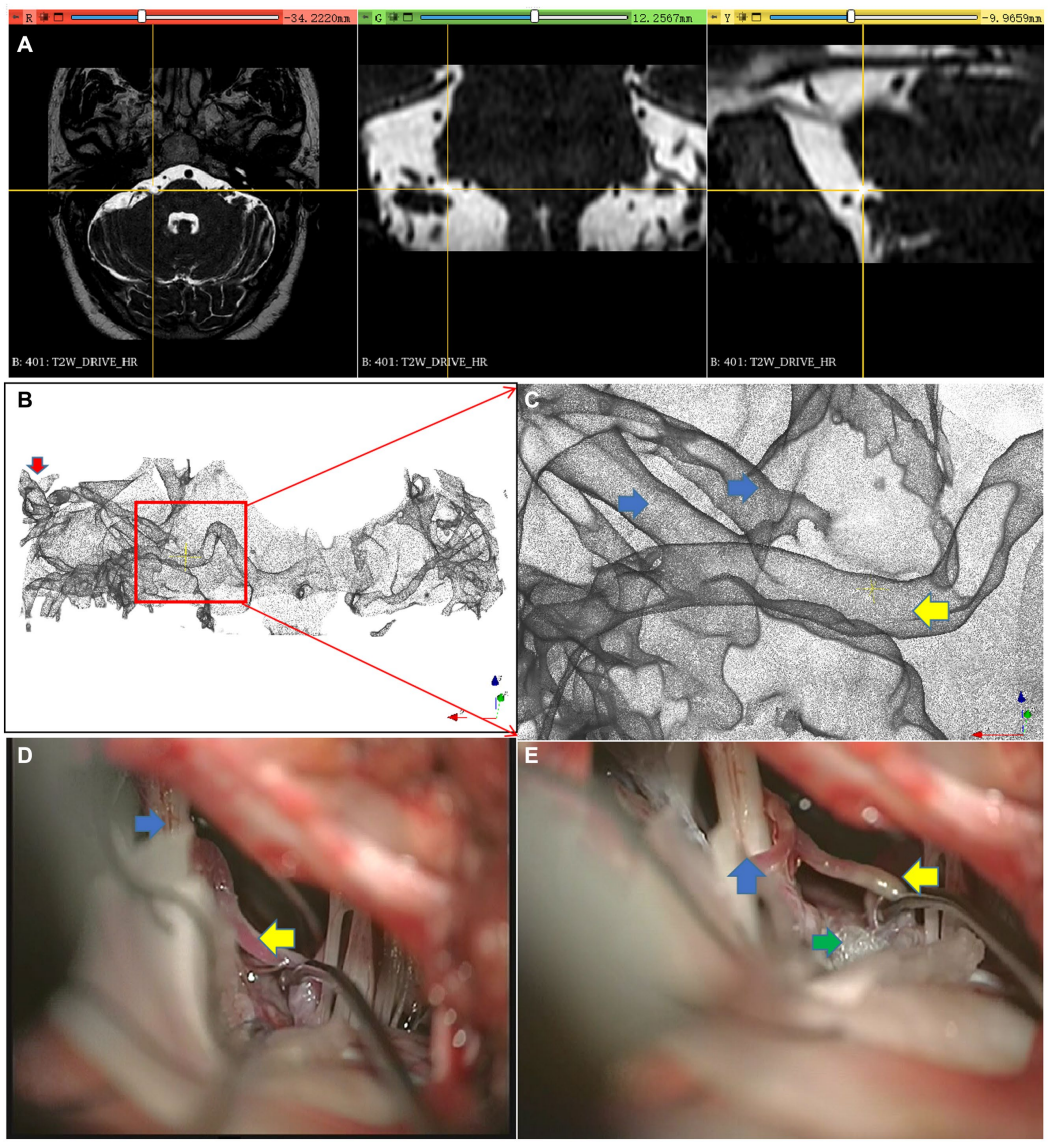
Single responsible vessel images. **(A)** Re-sliced image of the original image, **(B)** three-dimensional scene of NSIWR, **(C)** vessels and nerves within the REZ area; **(D,E)** microscopic images; Red arrow, Cochlea; Yellow arrow, Responsible vessel; Blue arrow, Facial nerve complex; Green arrow, Artificial material isolating vessels and nerves.

The patient experienced progressively worsening hemifacial spasm for 1 year without any identifiable trigger and was initially diagnosed with hemifacial spasm. Subsequently, the patient underwent MVD of the facial nerves. Pre-operative MR revealed a closely related blood vessel in the normal REZ, although its specific origin could not be determined (Figure 5). Using NSIWR, the cochlea was visualized and traced toward the facial nerve. In the REZ (Figures 5A–C), a single blood vessel originating from the PICA, which originated from the vertebral artery, was identified. The successful surgical (Figures 5D,E) intervention resulted in complete cessation of the spasms, achieving the desired level of treatment.

## 4 Discussion

The pre-operative evaluation of the responsible vessels in patients with HFS is significant for effective surgery and prognosis (Wang et al., 2020; Hughes et al., 2021). It can help determine surgical treatment methods for patients, such as the need for vertebral artery suspension and relocation to prevent recurrence after surgery (Dumot and Sindou, 2018; Han et al., 2021). Compared with previous studies (Zhou et al., 2012; Dolati et al., 2015; Yang et al., 2018), this study used non-segmented water outline rendering technology, which allows for a rapid 3D view of the vessels in the REZ. We identified its advantages compared to those using traditional image re-slicing techniques for identifying responsible vessels.

The inversion of water signals is beneficial for the visualization of nerves and vessels. By inverting water signals, the vessels and nerves in water can be displayed as “high-density” signals, similar to the imaging data of computed tomography scans, which is conducive for further realistic 3D rendering, making it easier to observe the relationship between nerves and vessels and identify the responsible vessels. The applicability of this method has also been confirmed in other studies, such as a study that inverted the 3D-FSE-T2WI sequence (Wang et al., 2020). The inverted data were in DICOM files with intervoxel gray-level gradients, which allowed the merging of opacity and shadows to provide realistic 3D sectional scans (Wang et al., 2020).

A water outline can effectively eliminate tissue signal interference and preserve the anatomical contours in potentially closed areas. In this study, a threshold interval was used to contour the neural vascular tissue in the inverted water signal, allowing for the removal of tissue structures enveloped by the water signal. This method successfully filtered out the interference from soft tissues in the brainstem, petrous bone, and slope direction. To the best of our knowledge, this is the first study to extract and observe neural vessels using outline rendering after inverting water signals using MRI.

Although some researchers have used multi-modal fusion technology to display the relationship between vessels and nerves (Guo et al., 2012; Brinzeu et al., 2018; Hastreiter et al., 2022), this approach requires registration and increases the scanning time, among other limitations (Donahue et al., 2017). Alternatively, certain researchers have employed flipped MRI image pixels and gray gradient rendering to enhance the accuracy of identifying responsible vessels (Wang et al., 2020). However, this method requires specific software and does not adequately separate the tissue occlusions in front of the brainstem.

Segmentation is a common method used in medical imaging to obtain ROIs by labeling and binarizing them as 1 or 0 (Khan et al., 2018). However, manual segmentation is a time-consuming and laborious process for segmenting the facial nerve, blood vessels, and REZ of the brainstem using MRI. Although this method has been extensively studied and the technology is relatively mature, it is rarely used in clinical practice because of the limited time and energy requirements of medical workers (Kasiri et al., 2014; Teton et al., 2019). Rendering is another 3D imaging method that can cross pixels with high-speed and realistic imaging characteristics. This saves a significant amount of time without relying on segmentation. For example, by using rendering to image nerves and blood vessels, we found that it not only achieved the segmentation effect but also had a significant impact on the clinical and surgical process, achieving the purpose of combining diagnosis and treatment based on imaging.

Fast and automatic reconstruction will become the preferred diagnostic method for doctors in the future (Donahue et al., 2017; Teton et al., 2019; Wang et al., 2020). The method used in this study can be completed within a short time. First, it avoids the need for 3D reconstruction of the entire brain, which allows it to be completed in a short time and is less dependent on computer memory. Second, only a single MR sequence can be used for 3D visualization, which helps achieve the reconstruction of neural vessels and avoids the time-consuming disadvantages of multi-modal techniques. Finally, the rendering method used in this study does not use cross-pixel pseudocolor rendering, which reduces the graphics card requirements and accelerates the process. In the future, the method used in this study can be automated, which will significantly increase its speed and reduce its usage thresholds.

Accurate diagnostic results can clearly guide clinical surgery regarding the responsible vessels (Brinzeu et al., 2018). Our research results indicate that there is a high consistency between non-segmented inverted water wheel rendering and the intraoperative view, which has obvious advantages over the traditional two-dimensional observation method of re-slicing. Further subgroup analyses suggested that it had significant advantages in multi-vessel reconstruction. In the evaluation of the three-dimensional reconstruction effect, we found that it also had a good effect on the reconstruction of small blood vessels. The reconstruction of nerves and vascular tissues is challenging when tracing their sources. In the evaluation of three-dimensional reconstruction, we found that the lymphatic fluid in the cochlea could achieve stable, high-quality reconstruction, and the spiral cochlea also had a high recognition ability in the three-dimensional structure. This evaluation also suggests that it has a positive effect on the reconstruction of nerves and blood vessels, especially on the small basal artery branches. To trace the blood vessels and nerves, we first tracked the facial nerve from the cochlea, located the REZ, examined the blood vessels in the REZ, and then traced the location of the blood vessels along the basal or vertebral artery, which helped us to make a quick standardized and accurate diagnosis.

Tracking blood vessels based on a two-dimensional re-slicing image is challenging when multiple vessels exist (Kasiri et al., 2014). When there are multiple culprit vessels, the blood vessels will overlap with nerves in the plane view. In contrast, the 3D rendering view used in this study allowed for a comprehensive understanding of the shape of the blood vessels to separate each culprit vessel. Our results confirmed this finding.

This study had some limitations. First, the nerves, arteries, and veins could not be separated directly; however, blood vessels could be identified by tracing them to the root of the basal or vertebral artery. Second, we did not compare images obtained with different scanning parameters, such as 1.5 T and 3.0 T. Therefore, it is currently unclear whether these different parameters would result in inconsistencies. Finally, the sample size was small, which could have affected our results. Therefore, further studies with larger sample sizes are needed to verify our findings.

This study encompassed clinical imaging observations and proposed a hypothesis for inverted water imaging. Through software development, the technique was applied for clinical validation, demonstrating that the non-segmented inverted contour rendering technique can visualize the relationship between blood vessels and nerves in a 3D scene. This would not only help doctors clarify blood vessel deformities in the REZ but would also make it easier to select surgical methods based on the degree of bending and winding of blood vessels.

## 5 Conclusion

This study reported a novel, fast, NSIWR method applied for hemifacial spasm. The results showed that the method could improve the accuracy of detecting the responsible vessels, especially in the case of multiple vessels. The evaluations by doctors indicated that this method also has better effects on the reconstruction of the cochlea and small blood vessels. However, the relatively small sample size of this study was a limitation. Therefore, future, large randomized controlled studies should be conducted to further validate our findings.

## Data availability statement

The original contributions presented in the study are included in the article/Supplementary material, further inquiries can be directed to the corresponding authors.

## Ethics statement

The studies involving humans were approved by Ningbo NO.2 Hospital Ethics committee. The studies were conducted in accordance with the local legislation and institutional requirements. Written informed consent for participation was not required from the participants or the participants’ legal guardians/next of kin in accordance with the national legislation and institutional requirements. Written informed consent was obtained from the individual(s) for the publication of any potentially identifiable images or data included in this article.

## Author contributions

LZ: Writing – original draft, Writing – review & editing. ZJin: Writing – review & editing. ZW: Writing – review & editing. ZJia: Writing – review & editing. XY: Writing – review & editing.
