# Supplementary material for: A novel non-segmented inverted water outline rendering method can improve the tracking of responsible blood vessels for hemifacial spasm
Source: Front Neurosci. 2024 Jan 30;18:1296019. doi: 10.3389/fnins.2024.1296019 (PMC10861737; doi:10.3389/fnins.2024.1296019)
Supplement: Supplementary file 1 [file Table_1.DOCX]

| Patient | Age(years) | Gender | Side | History(years) |
| --- | --- | --- | --- | --- |
| 1 | 56 | F | R | 2 |
| 2 | 43 | M | R | 1 |
| 3 | 55 | F | R | 3 |
| 4 | 60 | M | R | 2 |
| 5 | 73 | F | L | 1 |
| 6 | 38 | M | L | 2 |
| 7 | 46 | F | R | 0.4 |
| 8 | 44 | F | L | 1.5 |
| 9 | 56 | F | L | 4 |
| 10 | 65 | M | L | 3 |
| 11 | 43 | M | R | 5 |
| 12 | 62 | F | R | 10 |
| 13 | 56 | F | R | 4 |
| 14 | 52 | M | R | 4 |
| 15 | 51 | F | R | 3 |
| 16 | 48 | M | R | 3 |
| 17 | 73 | M | L | 2 |
| 18 | 68 | F | L | 2 |
| 19 | 47 | F | R | 2 |
| 20 | 45 | M | R | 2 |
| 21 | 58 | F | L | 1.5 |
| 22 | 54 | F | R | 2.5 |
| 23 | 44 | M | L | 4 |
| 24 | 45 | F | R | 3.5 |
| 25 | 58 | M | R | 6 |

**S Table-1 General patient data**

**S Table-1 The evaluation of the reconstruction effect**

| Case NO. | Reconstruction Quality Rating | | | | | Total Score |
| --- | --- | --- | --- | --- | --- | --- |
|  | Cochlea | Facial & Vestibulocochlear nerve | REZ | vertebral & basilar artery | Pica/ Aica |  |
| 1 | 2 | 2 | 2 | 2 | 2 | 10 |
| 2 | 2 | 2 | 2 | 2 | 2 | 10 |
| 3 | 2 | 2 | 2 | 1 | 2 | 9 |
| 4 | 2 | 2 | 2 | 2 | 2 | 10 |
| 5 | 2 | 2 | 2 | 2 | 2 | 10 |
| 6 | 2 | 2 | 2 | 2 | 2 | 10 |
| 7 | 1 | 1 | 1 | 1 | 1 | 5 |
| 8 | 2 | 1 | 1 | 2 | 1 | 7 |
| 9 | 2 | 2 | 2 | 2 | 2 | 10 |
| 10 | 2 | 2 | 2 | 2 | 1 | 9 |
| 11 | 2 | 2 | 2 | 2 | 2 | 10 |
| 12 | 2 | 2 | 2 | 2 | 2 | 10 |
| 13 | 2 | 2 | 2 | 1 | 2 | 9 |
| 14 | 2 | 2 | 1 | 2 | 2 | 9 |
| 15 | 2 | 2 | 2 | 2 | 2 | 10 |
| 16 | 2 | 2 | 2 | 2 | 2 | 10 |
| 17 | 2 | 2 | 2 | 1 | 1 | 8 |
| 18 | 2 | 2 | 2 | 2 | 2 | 10 |
| 19 | 2 | 2 | 2 | 2 | 2 | 10 |
| 20 | 2 | 2 | 2 | 2 | 1 | 9 |
| 21 | 2 | 2 | 2 | 1 | 2 | 9 |
| 22 | 2 | 2 | 2 | 2 | 2 | 10 |
| 23 | 2 | 2 | 2 | 2 | 2 | 10 |
| 24 | 1 | 1 | 1 | 1 | 1 | 5 |
| 25 | 2 | 2 | 2 | 2 | 2 | 10 |
| Total Score | 48 | 47 | 46 | 44 | 44 | 229 |
|  | (excellent:92%) | (excellent:88%) | (excellent:84%) | (excellent:76%) | (excellent:76%) |  |

1. **MR Information**

MR Instrument: Philips Achieva Nova Dual 3.0 T superconducting magnetic resonance imaging system with an 8-channel phased array head coil.

Scanning parameters: 3D-T2-DRIVE sequence setting parameters: FOV is 130mm×130mm, layer thickness is 0.7mm, number of layers is 60, reconstruction matrix is 512×512, TR is 1500ms, TE is set to 250ms, flip angle is 90°, the average acquisition times are 2, and the scan time is set to 6min15s
